# Does older age justify chlorambucil control arms for chronic lymphocytic leukemia clinical trials: a SEER-Medicare analysis

**DOI:** 10.1038/s41375-023-01915-y

**Published:** 2023-05-23

**Authors:** Akiva Diamond, Long Vu, Wyatt P. Bensken, Siran M. Koroukian, Paolo F. Caimi

**Affiliations:** 1 Dan L Duncan Comprehensive Cancer Center at Baylor St. Luke’s Medical Center, Houston, TX USA; 2 Population Cancer Analytics Shared Resource, Case Comprehensive Cancer Center, Cleveland, OH USA; 3 Department of Population and Quantitative Health Sciences, School of Medicine, Case Western Reserve University, Cleveland, OH USA; 4 Cleveland Clinic Taussig Cancer Center, Cleveland, OH USA

**Keywords:** Chronic lymphocytic leukaemia, Chemotherapy

## To the Editor:

Ibrutinib has demonstrated prolonged responses in chronic lymphocytic leukemia (CLL) and changed the treatment paradigm from short course chemo-immunotherapy to continuous oral treatment. The FDA approved ibrutinib as initial therapy for CLL in March 2016, based on results of the RESONATE-2 trial [1]. Treatment naive CLL patients ≥65 years were randomly assigned to ibrutinib or up to 12 treatment cycles of chlorambucil. Ibrutinib was superior, with a higher response rate, longer progression-free survival (PFS) and overall survival. The iLLuminate study enrolled participants ≥65 years or patients with coexisting conditions, and compared ibrutinib plus obinutuzumab versus chlorambucil plus obinutuzumab, showing significantly longer PFS in the ibrutinib arm.

Enrollment to these trials was limited to patients 65 years or those with comorbidities, yet the results led to adoption of ibrutinib across age groups [2]. However, equipoise of using chlorambucil as a control arm has been questioned [3]. It was acknowledged that patients younger than 75 achieved significantly longer PFS with bendamustine than chlorambucil [1]. In the Connect CLL registry, an observational cohort of CLL patients in the United States, only 4.5% of patients received frontline chlorambucil in the years preceding the RESONATE-2 trial [3]. The recently published GLOW trial, a phase 3 trial of fixed-course venetoclax and ibrutinib compared to chlorambucil and obinutuzumab, has garnered considerable debate regarding usage of a chlorambucil-based control arm [4].

The RESONATE-2, iLLuminate and GLOW trials considered chlorambucil as adequate control among patients aged 65 or older, but little is known about chlorambucil as part of routine clinical care at the time of these trials. Our aims were to retrospectively evaluate practice patterns in SEER-Medicare data during the same timeframe as the frontline ibrutinib clinical trials and describe characteristics of CLL patients prescribed chlorambucil during this period.

Using Surveillance, Epidemiology, and End Results Program (SEER) data linked with Medicare claims we identified 17,114 patients diagnosed with incident CLL between 2007 and 2015. We required full coverage in Medicare Parts A, B, and D from the start of enrollment through their entire follow-up time. We included patients 66 years of age or older at diagnosis to allow for a one-year lookback period, during which we identified Elixhauser comorbidities. Our final analytic cohort included 4958 patients. Advanced stage disease was flagged by the presence of anemia or thrombocytopenia. Chemotherapy treatment was identified using generic names as well as Healthcare Common Procedure Coding System (HCPCS) codes. A claims-based frailty index was calculated for each patient, and were categorized as non-frail, pre-frail, and frail [5, 6].

We looked at first line treatment rates of chlorambucil and other chemotherapy agents per year, and during three different time periods: (1) before the RESONATE-2 and iLLuminate studies opened (Jan 2008 to Feb 2013); (2) during the enrollment period of these studies (Mar 2013 to Oct 2015); and (3) after enrollment completion (Nov 2015 to Dec 2016).

Of the 4958 patients identified, 1389 (28.0%) received frontline therapy during the study period. Among these treated patients, median age at diagnosis was 76 years (IQR: 71–82). Advanced stage disease was present in 51% of patients and 27.8% had 3 or more comorbidities (Table 1).

**Table 1:** Baseline characteristics of the analytic cohort.

	Overall, *n* = 1389 (%)	RITUX, *n* = 365 (%)	RITUX + BENDA, *n* = 279 (%)	CHLOR, *n* = 198 (%)	FCR/FR, *n* = 168 (%)	IBRUT, *n* = 109 (%)	CYCLO + OTHER, *n* = 83 (%)	CHLOR + OBINU, *n* = 54 (%)	OTHER, *n* = 133 (%)
Age at diagnosis, Median [IQR]	76 [71, 82]	79 [73, 85]	74 [70, 79]	79 [74, 85]	72 [69, 77]	76 [71, 81]	74 [70, 79]	74 [70, 80]	77 [71, 81]
Age at treatment initiation, Median [IQR]	77 [73, 83]	80 [74, 86]	76 [72. 80]	80 [75, 86]	73 [70, 78]	77 [73, 84]	76 [71, 81]	76 [74, 82]	78 [73, 83]
Sex, female	620 (44.6)	169 (46.3)	114 (40.9)	108 (54.5)	63 (37.5)	45 (41.3)	37 (44.6)	28 (51.9)	56 (42.1)
Race									
White	1263 (90.9)	327 (89.6)	261 (93.5)	174 (87.9)	>157 (>93.5)	98 (89.9)	>75 (>90.4)	>49 (>90.7)	119 (89.5)
Other	126 (9.1)	38 (10.4)	18 (6.5)	24 (12.2)	<11 (<6.5)	11 (10.1)	<11 (<13.2)	<11 (<20.4)	14 (10.6)
Number of Elixhauser conditions									
0	437 (31.5)	94 (25.8)	99 (35.5)	56 (28.3)	55 (32.7)	36 (33.0)	18 (21.7)	26 (48.1)	53 (39.8)
1–2	566 (40.8)	145 (39.7)	116 (41.6)	75 (37.9)	80 (47.6)	49 (45.0)	40 (48.2)	>19 (>35.2)	>40 (>30.0)
3+	386 (27.8)	126 (34.5)	64 (22.9)	67 (33.8)	33 (19.6)	24 (22.0)	25 (30.1)	<11 (<20.4)	<40 (<30.0)
Advanced stage CLL	709 (51.0)	237 (64.9)	123 (44.1)	96 (48.5)	70 (41.7)	40 (36.7)	49 (59.0)	23 (42.6)	71 (53.4)
Fraility Index									
Non-Frail	147 (10.6)	32 (8.8)	33 (11.8)	<11 (<5.6)	28 (16.7)	15 (13.8)	<11 (<13.3)	>11 (>20.4)	11 (8.3)
Pre-Frail	932 (67.1)	237 (64.9)	187 (67.0)	>137 (>69.2)	118 (70.2)	71 (65.1)	>58 (>69.9)	32 (59.3)	90 (67.7)
Frail	310 (22.3)	96 (26.3)	59 (21.2)	50 (25.3)	22 (13.1)	23 (21.1)	<20 (<22.0)	<11 (<20.4)	32 (24.1)

Rituximab was the most common frontline treatment, prescribed to 26.3% of patients. Median age at first treatment with rituximab was 80 years (IQR: 74–86), 64.9% had advanced stage disease, and 34.5% had at least 3 comorbidities. The next most frequently prescribed regimens were bendamustine with rituximab (BR), followed by fludarabine and rituximab containing regimens (FCR/FR), with 20.1% and 12.2% of patients, respectively. Median age at first treatment for BR and FCR/FR respectively was 76 and 73 years (IQR: 72–80 and 70–78), 44.1% and 41.7% had advanced stage disease, and 22.9% and 19.6% had at least 3 comorbidities (Table 1).

**Fig. 1:**
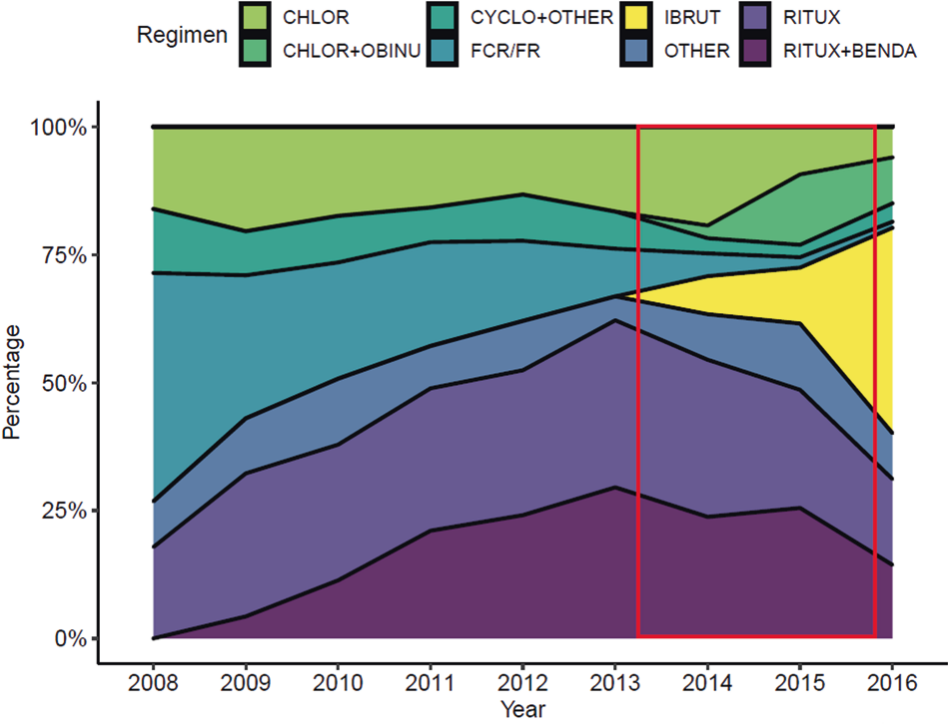
CLL treatment by year of initiation. Red inset represents the enrolment period of the RESONATE-2 and iLLuminate studies. CHLOR chlorambucil, CHLOR + OBINU chlorambucil and obinutuzumab, CYCLO + OTHER cyclophosphamide with other agents, including but not limited to doxorubicin and/or vincristine, FCR/FR fludarabine with cyclophosphamide and rituximab or fludarabine with rituximab, IBRUT ibrutinib, OTHER other single agent or combination chemotherapy agents, RITUX single agent rituximab, RITUX + BENDA rituximab with bendamustine.

There were notable treatment trends by year during the study period. FCR/FR was initially the most commonly prescribed treatment and steadily declined on a yearly basis (Fig. 1). In 2008, FCR/FR was prescribed in 44.6% of patients and decreased to 1.2% by 2016. Ibrutinib had a sharp increase from 7.4% in 2014, the year of its initial FDA approval (for relapsed CLL), to 40.1% by 2016. BR first saw an increase in 2010 (11.4%), peaked in 2015 (25.5%), and decreased again in 2016 (14.4%). Chlorambucil use remained relatively stable over time, with a shift in the last 3 years of the study period from single agent to usage in combination with obinutuzumab.

During the enrollment period of the ibrutinib versus chlorambucil clinical trials (Mar 2013 to Oct 2015), BR and rituximab monotherapy were the most common treatments prescribed (27.4% and 27.2%, respectively). Chlorambucil alone was prescribed in 15.0% of patients, while chlorambucil plus obinutuzumab was used as first-line treatment in 5.6% of CLL patients (Fig. 1).

During our total observation period (Jan 2008 to Dec 2016), fewer than 15% (14.3%) of patients were treated with chlorambucil and 3.9% with chlorambucil and obinutuzumab. Chlorambucil-treated patients had a median age of 79 years (IQR: 74–85) at time of diagnosis and 80 years (IQR: 75–86) at time of first treatment. Advanced stage disease was present in 48.9%, and 33.8% had at least 3 comorbidities. Only 5.1% of chlorambucil-treated patients were considered non-frail, compared to 8.7%, 16.7% and 11.8% of rituximab-, BR-, and FCR/FR-treated patients, respectively (Table 1).

During the period of the pivotal ibrutinib clinical trials, more patients 66 years and older were prescribed intensive chemotherapy (BR and FCR/FR) than chlorambucil. Chlorambucil- and single agent rituximab-treated patients were older, had higher frailty, and more comorbidities than BR and FCR/FR treated patients.

The iLLuminate study considered all patients over 65 to be unfit to receive FCR due to advanced age. Our data reveal that in the years prior to the trial, FCR was commonly used in this age group. The median age in iLLuminate was 71 years, while the median age of FCR treated patients in our dataset was 73 years (IQR: 70–78). The decrease in FCR and increase in BR use coincides with the initial presentation of the CLL2M trial data in 2009 which showed high response rates with BR [7].

Although ibrutinib was approved based on its superiority to chlorambucil, the prescription patterns in the years prior to its approval were characterized by low rates of chlorambucil, while the patterns following ibrutinib approval appear to have been characterized by decreasing rates of BR rather than a drop in chlorambucil prescription rates.

The median age of patients enrolled in RESONATE-2 was 72 years, while the median age of chlorambucil-treated patients in the SEER-Medicare dataset was 80 years, and 25.3% were considered at least mildly frail.

Between November 2015 and December 2016, chlorambucil and obinutuzumab were only prescribed in 10.9% of patients, compared to prescription rates of 14.7% for BR, and 34.1% for ibrutinib. BR was more commonly prescribed to older SEER-Medicare CLL patients prior to the GLOW study initiating enrollment in May of 2018 [4]. The median age in the GLOW trial was 71 years while the median age of BR-treated patients in the SEER-Medicare dataset was 74 years. Although both GLOW and RESONATE-2 were international trials, they included U.S. study sites, with the largest number of patients in the RESONATE-2 trial from the USA [1].

In this analysis, we show that single agent chlorambucil or rituximab use in the period 2008–2016 was limited to the oldest treated patient subgroups with the highest comorbidity burden. Prior to the GLOW study opening (2018), US practice patterns had already shifted away from chlorambucil and obinutuzumab, even among older patients. Fixed-duration BR was a regimen more representative of the treatment prescribed to CLL patients aged over 65 and treated in the USA at the time of trials evaluating ibrutinib, which guided its regulatory approvals.

Our data show that in the period when chlorambucil was used as a control arm in ibrutinib trials, CLL patients of comparable age were more commonly treated with more aggressive therapies. It is apparent that standard practice in the U.S. had largely phased out chlorambucil, restricting it to even older, more frail patients. Previous criticisms of the use of chlorambucil control arms for CLL studies appear to have validity [3]. Our findings show that trials using chlorambucil greatly influenced prescription patterns of more intense frontline CLL therapies, leading to a decrease in their use, before randomized trials demonstrating the superiority of ibrutinib over more intense chemoimmunotherapy [8, 9].

The overall positive trial results are likely a consequence of the high efficacy and safety of ibrutinib. However, recognizing potential hindsight bias, it could be stated that chlorambucil was an obsolete control for the population studied, and the results of trials including it were applied in clinical practice beyond their reported findings. For future clinical trials, it would be important to use the common standard care practice as control arm. The choice of control treatment can significantly impact the clinical relevance and validity of the trial’s results, especially in an era of rapidly evolving treatments.

## Data Availability

The data that support the findings of this study are available from the National Cancer Institute. The collection of cancer incidence data used in this study was supported by the California Department of Public Health pursuant to California Health and Safety Code Section 103885; Centers for Disease Control and Prevention’s (CDC) National Program of Cancer Registries, under cooperative agreement 1NU58DP007156; the National Cancer Institute’s Surveillance, Epidemiology and End Results Program under contract HHSN261201800032I awarded to the University of California, San Francisco, contract HHSN261201800015I awarded to the University of Southern California, and contract HHSN261201800009I awarded to the Public Health Institute. The ideas and opinions expressed herein are those of the author(s) and do not necessarily reflect the opinions of the State of California, Department of Public Health, the National Cancer Institute, and the Centers for Disease Control and Prevention or their Contractors and Subcontractors.
